# Racing Fast and Slow: Defining the Tactical Behavior That Differentiates Medalists in Elite Men's 1,500 m Championship Racing

**DOI:** 10.3389/fspor.2019.00043

**Published:** 2019-10-14

**Authors:** Gareth N. Sandford, Benjamin T. Day, Simon A. Rogers

**Affiliations:** ^1^School of Kinesiology, University of British Columbia, Vancouver, BC, Canada; ^2^Canadian Sport Institute-Pacific, Victoria, BC, Canada; ^3^Athletics Canada, Ottawa, ON, Canada; ^4^High Performance Sport New Zealand, Auckland, New Zealand; ^5^School of Health & Human Science, Southern Cross University, Lismore, NSW, Australia; ^6^Australian Institute of Sport, Canberra, ACT, Australia

**Keywords:** performance, middle-distance running speed, coaching, tactics, strategy

## Abstract

**Background:** 1,500 m running has long been a blue ribbon event of track championship racing. The eventual medalists employ common tactical behaviors such as a fast sustained pace from the start (gun-to-tape), or, slow initial laps that precede a precisely timed race kick. Before the kick, there are positional changes caused by surging, that can go uncharacterized. The inter-relationship of surge events, tactical positioning, and kick execution may have important implications for eventual medal winning outcomes and require further definition.

**Methods:** In a randomized order, three middle-distance running experts were provided publically available video (YouTube) of 16 men's 1,500 m championship races across, European, World and Olympic championships. Each expert determined the occurrence of surges (defined as any point in the 1,500 m after the first 300 m where an athlete repositions by ≥3 places; or noticeably dictates a raise in the pace from the front) and the race kick. Following a second level verification of expert observations, tactical behaviors (quantity and distance marker within each race) mean distance from the finish were compared between fast (≤3:34.00, *n* = 5), medium (>3:34.00– ≤3:41.99, *n* = 7) and slow (≥3:42.00, *n* = 4) race categories.

**Results:** Before the race kick, there were more surges in slow (5 ± 1.7, mean ±90% confidence limits) vs. fast races (1 ± 0.4, very large difference, *very likely*). The final surge before the race kick occurred earlier in fast (704 ± 133 m from the finish) vs. medium (427 ± 83 m, large difference, *most likely*), and slow races (370 ± 137 m, large difference, *most likely*). At initiation of the race kick in fast races, large positional differences were found between eventual gold (2 ± 1.2; *likely*) and silver (2.2 ± 1.6; *likely*) vs. bronze medalists (4.4 ± 1.2). In slow races, positional differences were *unclear* between eventual gold (4.3 ± 4.7), silver (4.8 ± 4.8) and bronze medalists (5.3 ± 1.5). Regardless of category, the race kick occurred on the last lap, with *unclear* differences between fast 244 ± 92 m medium 243 ± 56 m and slow 236 ± 142 m races.

**Conclusions:** Presenting tactical behaviors by race categorization (slow, medium, fast race times), provides a novel understanding of the nuance of racing tactics. The present findings highlight the importance of considering within race athlete decision making across multiple-race scenarios during championship preparation.

## Introduction

It is well-established across the middle-distance running literature that the final surge, more commonly known as the finishing kick, differentiates medalists in championship racing of the 1,500 m (Thiel et al., 2012; Mytton et al., 2015; Aragón et al., 2016; Casado and Renfree, 2018; Hanley et al., 2018). 1,500 m championship racing is akin to a game of chess, whereby an athlete must jostle for a favorable tactical position, ready to execute the “kick” at the opportune moment.

The current literature, whilst highlighting the concept of the “race kick” in the 1,500 m, is limited in methodology in several areas. Firstly, jostling for position in the early phases of the race is often overlooked and may require an element of surging that has bioenergetic consequences on an athlete's ability to kick (Fukuba and Whipp, 1999; Jones and Whipp, 2002). Importantly, these mid-race surges before the kick (i.e., up to and including the final surge), position the athlete to strike and are a critical piece of tactical execution (Hanley et al., 2018). Secondly, tactical assessments in the literature are often limited to analysis between intermediate race position and the probability of qualifying/progressing through rounds, as identified from sector splits (commonly only to 100 or 200 m sectors) (Renfree et al., 2014; Mytton et al., 2015; Casado and Renfree, 2018; Hanley et al., 2018). Whilst splits and subsequent velocity profiles are useful for general race characterization, an athlete's intermediate tactical position in relation to surges or the “kick” may occur between the resolution of the venues timing splits. With sports federation funding primarily determined by medal outcomes, providing further resolution on the medalists underpinning tactical behavior is of critical performance interest for coaches, athletes and sport performance staff.

Current literature on “the kick” in the 1,500 m is typically assessed from a one off championship (Renfree et al., 2014; Casado and Renfree, 2018), or in cases assessing multiple championships, an assumption is made that finals of varying finish time, whether that be fast, medium, or slow, result in the similar tactical execution (Mytton et al., 2015; Hanley et al., 2018). This assumption may be a misrepresentative interpretation of the tactical behavior required to win across a broad set of tactical scenarios. Athletes must be prepared for all eventualities on race day, which could range from gun-to-tape to sit and kick style racing (Fukuba and Whipp, 1999; Jones and Whipp, 2002). For example, the 2016 Rio de Janeiro Olympics men's 1,500 m final finish time was noted as “slow” (3:50.00) whereas the 2017 London World Championship was won in a markedly “faster” time (3:33.61). Thus, it is imperative for tactical positioning throughout the 1,500 m to be assessed with these different “fast,” “medium,” and “slow” pace categories in mind, and consider differences in bioenergetic demand and effort distribution. Ultimately, the difference in strategies required and the implications for coach and athlete preparation, training and race execution may be substantial.

Therefore, the current study aimed to answer the following questions in male 1,500 m races across European, Olympic and World Championships, between 2000 and 2017: (i), at what distance from the finish are race surges and the race finishing kick initiated and where were the eventual medalists positioned at these key points in the race? (ii), how do these outcomes differ in fast, medium, and slow pace race categories?

## Methods

The tactical positioning of men's 1,500 m medalists during surges and the kick was assessed using readily available footage from YouTube of the following elite athletics championships where medals are the priority, not finishing times or seasons best time: European Championships 2012, 2014, 2016, World Championships 2001, 2003, 2007, 2009, 2011, 2013, 2015, 2017, and Olympic Games 2000, 2004, 2008, 2012, 2016.

The term “kick” may be looked at from two perspectives. First, in reference to the “race kick” which globally characterizes the kick event within the race, defined herein as, “a one-time landmark moment in the race where the first athlete makes the races' first decisive and final break from the pace as a sustained pursuit for the finish line.” Furthermore, there will be other athletes who execute their kick after this point (i.e., when each individual decides to utilize their remaining energetic resources), these are tactical responses to the first athlete who initiated the race kick (Renfree et al., 2015; Hettinga et al., 2017; Konings and Hettinga, 2018), thus these instances will be referred to herein as the “athlete kick.” For clarity, the race kick will be the emphasis of the present investigation, with the athlete kick definition provided as a basis for clarity of interpretation, and opportunity for future investigation. Race surges often go uncharacterized, therefore, we herein defined in-race surges as “any point in the 1,500 m after the first 300 m where an athlete repositions by ≥3 places; or noticeably dictates a raise in the pace from the front (i.e., they are in the first 3 places)”.

To overcome the limitation of 100 m sector resolution on tactical behaviors in previous studies, three independent middle-distance experts (12–20 years' experience working in the sport) analyzed the 16 race videos in a randomized order (Altman and Bland, 1999), following a familiarization trial of a race not included in the study (*n* = 1). To identify the medalists (top 3 finishers), experts were provided with the name, nationality, finishing position, finishing time, bib number and singlet color of each athlete. For each race, experts recorded the following: (a) the distance from the finish line that surges (i.e., total count) and the race kick were deemed to occur [sometimes this was not a medalist and were requested to give their expert judgement to the nearest 10 m)]; (b) the position (1st, 3rd, 5th etc.) of the eventual medalists during the respective surges and the race kick in (a). When athletes were side by side, the person on the inside rail was deemed in the higher position as running one lap in lane 2 adds 7 m extra race distance (Jones and Whipp, 2002).

Experts were instructed to watch videos without commentary, and permitted to stop or replay the video as often as required to accurately provide their observations. In addition, experts were asked not to look at online results of the race (IAAF website etc.) until after they had watched the race and finalized their answers. Once the expert results were collected, the primary and third author provided further verification. Whereby, no additional surges were introduced, but observations of experts on specific surge or race kick distances from the finish were confirmed using track landmarks (e.g., the 400 m finish line).

Races were characterized with the following rationale: “Fast pace” defined as race finishing times ≤ 3:34.00 (*n* = 5). Whereby, in the last 5 years <20 athletes per year have run faster than 3:34.00 from any nation. With a field allocation of 12 athletes in an IAAF World Championships or Olympic final, 3:34 was chosen as a pace that would be deemed fast by all eventual finalists. “Medium pace” (3:34.01–3:41.99) was selected as 3:41.99 is the United States of America Track and Field Trial “B” standard to compete at their National Championships (*n* = 7) and “Slow pace” were times ≥3:42.00 (*n* = 4).

## Analysis

Data are presented as means and 90% confidence limits (CL) unless otherwise stated. Mean differences in distance from the finish (m) and tactical position of the medalists at initiation of surges and the race kick, alongside number of surges before the race kick were assessed between fast, medium and slow pace races using magnitude based inferences (Hopkins et al., 2009). Comparison between means were calculated using a spreadsheet (Hopkins, 2007). The threshold values for effect size (ES) statistics were ≤0.2, trivial if there is overlap of 90% CL on positive or negative effect) or unclear (if 90% CL overlaps both positive and negative effect), ≥0.2 (small), >0.6 (moderate), >1.2 (large), and >2.0 (very large) (Batterham and Hopkins, 2006). The smallest worthwhile change was calculated as the standard deviation of the variable “e.g., Tactical position at the race kick for all race categories,” multiplied by the ES. Probabilities were used to make a qualitative probabilistic inference about the true differences. The scale for interpretation was: <0.5%, most unlikely, almost certainly not; 0.5–5%, very unlikely; 5–25%, unlikely, probably not; 25–75%, possibly; 75–95%, likely, probably; 95–99.5%, very likely; >99.5%, most likely, almost certainly (Hopkins et al., 2009).

## Results

**Figure 1:**
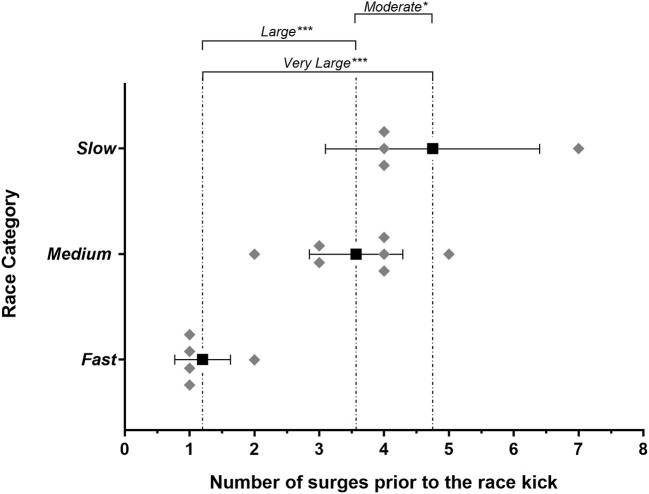
Number of surges before the race kick within each individual *fast* (*n* = 5), *medium* (*n* = 7), and *slow* (*n* = 4) race analyzed, and the mean (90% CL) for each category in the solid square plots. Brackets indicate the magnitude differences between means of each of the three race categories with the probability of each difference indicated as *possibly substantial difference, **likely substantial difference, ***very likely substantial difference, and ****most likely substantial difference.

**Figure 2:**
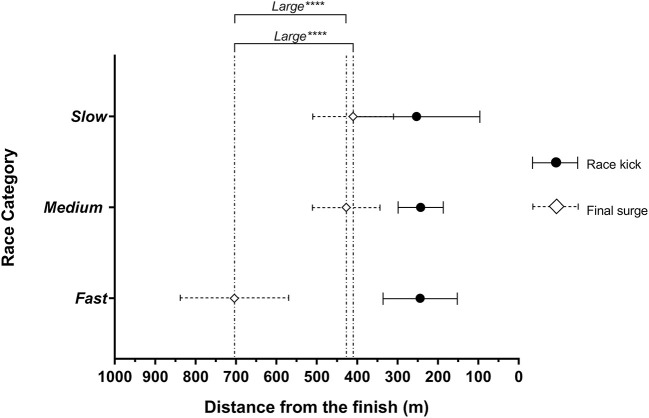
Mean distance from the finish line of the reported final surge and race kick across fast (*n* = 5), medium (*n* = 7), and slow (*n* = 4) race categories. Brackets indicate the substantial magnitude differences between means of each final surge across the three race categories. The probability of each difference is indicated as *possibly substantial difference, **likely substantial difference, ***very likely substantial difference, and ****most likely substantial difference. Unmarked comparisons showed unclear differences. Error bars represent the 90% confidence limits.

Mean and individual finish times for fast (*n* = 5), medium (*n* = 7), and slow (*n* = 4) categories are shown in Table 1. Before the race kick, there were more surges in slow (5 ± 1.7) vs. fast races (1 ± 0.4, very large difference, *very likely*). There were more surges in both slow vs. medium races (1.4 ± 1.7 moderate difference, *possibly*) and medium vs. fast races (2.4 ± 0.5 large difference, *very likely*), all Figure 1. The final surge before the kick occurred substantially earlier in a fast paced 1,500 m (704 ± 133 m from the finish) compared to medium (427 ± 83 m, large difference, *most likely*), and slow 1,500 m races (370 ± 137 m, large difference, *most likely*), as shown in Figure 2. Regardless of race category examined, the race kick occurred within the last 400 m (Figure 2), with *unclear* differences between race kick distance from the finish in the slow 236 ± 142 m, medium 243 ± 56 m and fast 244 ± 92 m races.

**Table 1:** Men's 1,500 m championship race finishing times in their slow, medium, and fast categories.

**Pace category**	**Event**	**Year**	**Winning time (m:ss.ss)**
“*Fast”*	WC (London)	2017	3:33.61
	OG (Beijing)	2008	3:32.94
	WC (Paris)	2003	3:31.77
	WC (Edmonton)	2001	3:31.68
	OG (Sydney)	2000	3:32.07
**Mean ± CL**			**3:32.21 ± 0:01.65**
“*Medium”*	WC (Beijing)	2015	3:34.40
	WC (Moscow)	2013	3:36.28
	OG (London)	2012	3:34.08
	WC (Daegu)	2011	3:35.69
	WC (Berlin)	2009	3:35.93
	WC (Osaka)	2007	3:34.77
	OG (Athens)	2004	3:34.18
**Mean ± CL**			**3:35.05 ± 0:01.12**
“*Slow”*	OG (Rio de Janeiro)	2016	3:50.00
	EC (Amsterdam)	2016	3:46.65
	EC (Zurich)	2014	3:45.60
	EC (Helsinki)	2012	3:46.20
**Mean ± CL**			**3:47.11 ± 0:03.24**

*WC, IAAF world championships; OG, olympic games; EC, European athletics championships; Fast, ≤3:34.00; Medium, 3:34.00–3:41.99; Slow, ≥3:42.00*.

**Figure 3:**
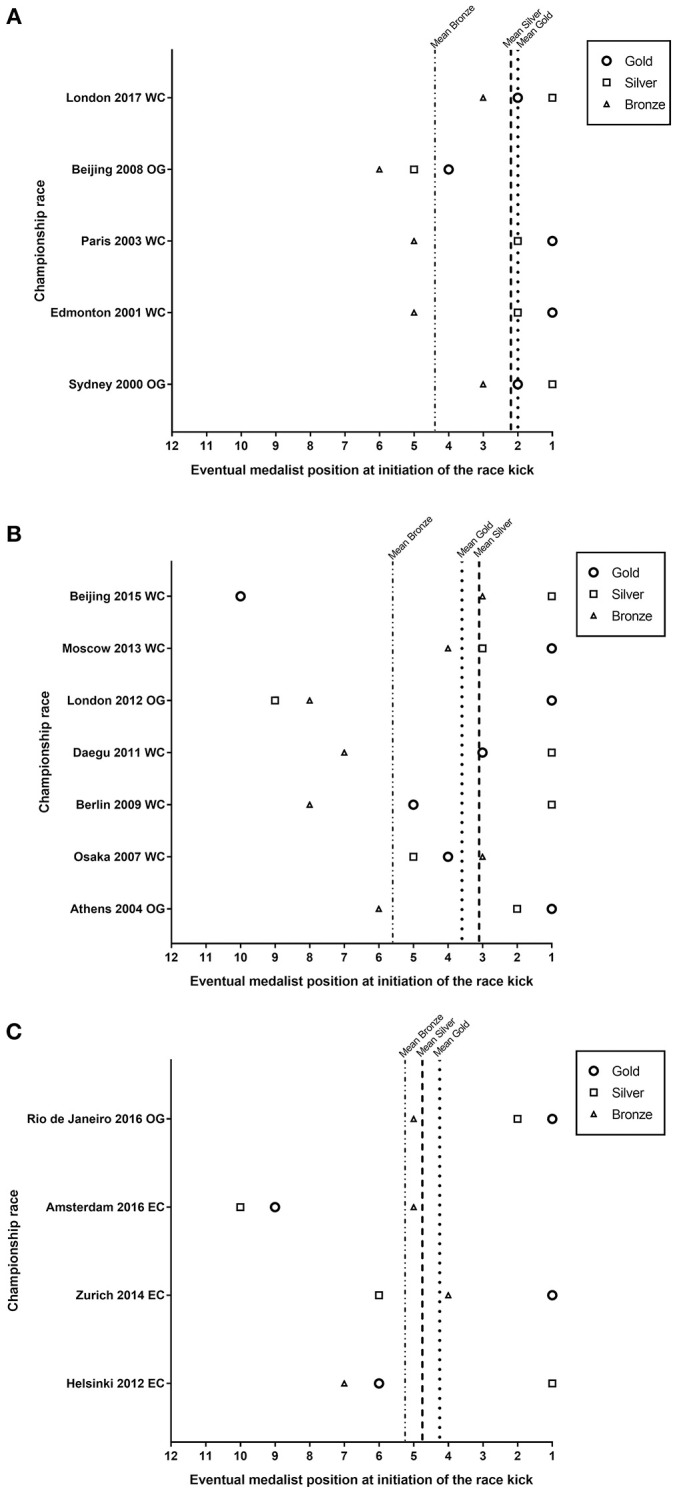
Positions of all eventual medalists at the initiation of the race kick in each individual race from the fast **(A)**, medium **(B)**, and slow **(C)** race categories. WC, IAAF world championships; OG, olympic games; EC, European athletics championships.

At initiation of the race kick, eventual medalists were in higher race positions in fast races (top 3 ± 1.2), than in slow paced races (top 5 ± 2.3, moderate difference, *possibly*). Specifically, within fast races, there was a large difference (*likely)* in the tactical position of the eventual gold (2 ± 1.2) and silver (2.2 ± 1.6) medalists vs. the eventual bronze medalist (4.4 ± 1.3), see Figure 3A. In medium pace races (Figure 3B), there were *unclear* differences in the tactical position of eventual gold (3.6 ± 2.4) and silver medalist (3.1 ± 2.2), however eventual bronze medalists (5.6 ± 1.6) were in lower positions at the race kick than silver (moderate difference; *likely*), and gold medalists (moderate difference; *possibly*). Across slow races (Figure 3C), there were *unclear* differences in the tactical position at initiation of the race kick between eventual gold (4.3 ± 4.7), silver (4.8 ± 4.8) and bronze medalists (5.3 ± 1.5).

## Discussion

The present study is the first in elite male 1,500 m runners to report the concurrent tactical positioning of eventual medalists alongside surge events and the race kick using a novel methods approach of fast, medium, and slow race category analysis. It revealed that substantially different tactical approaches are undertaken in different paced championship races. Specifically, on average more surges occur before the race kick in slow races, compared to fast and medium races (Figure 1). Surge events are most variable between slow races (Figure 1), and the final surge before the race kick occurs substantially earlier in fast vs. medium and slow races (Figure 2). Consistent with previous observations (Thiel et al., 2012; Renfree et al., 2014; Aragón et al., 2016; Casado and Renfree, 2018; Hanley et al., 2018), we show the race kick as a key tactical behavior, that interestingly occurred on the last lap irrespective of the race category (Figure 2). For the first time we highlight the important differences in the role of tactical position at initiation of the race kick between fast, medium and slow races. To win the gold medal in fast races, athletes at the race kick on average were shown to be in the top 2 positions, vs. top 4 in medium and slow races, respectively (Figures 3A–C). Importantly in slow pace races, there was no substantial difference in tactical position at the race kick of all eventual medalists (Figure 3C). Together these findings have numerous methodological implications for future research studies alongside training and tactical preparation for coaches and athletes toward championship racing.

An athlete has limited metabolic resources to portion across the course of a race (Fukuba and Whipp, 1999) and will self-regulate the use of this resource according to environmental feedback (weather conditions, opponents position, surging) to put themselves in an eventual medal-winning position. This demonstrates a concept known as affordance-weighted decision making (Hettinga et al., 2017). In a slow race, all athletes typically operate at a relatively low rating of perceived exertion in the early laps (~critical velocity; Sandford et al., 2019), and therefore everyone is vulnerable to a surge attack. This leads to an increase in tension about tactically being in a “good position,” which may in part explain the increase in the number of sometimes erratic of mid-race surges (Figure 1; Konings and Hettinga, 2018). Developing in-race positional awareness, subsequent tactical agility and affordance-weighted decision making becomes increasingly important the slower the race, with surges continuing to happen very close to the race kick in both medium and slow races (Figure 2). In contrast, a fast paced race has a higher perception of effort and given physiological strain from the start line (Tucker et al., 2006). Seemingly this reduces the tactical decision making element of having to be in position (i.e., fewer surges; Figure 1), as attention is focused on trying to stay with the fast pace, akin to that of a world record attempt (Tucker et al., 2006; Foster et al., 2014) (i.e., if an athlete cannot stay with a fast early pace, they will not be in the medal-winning positions, irrespective of tactics). In support of this explanation, Figure 2 also shows that the final surge before the race kick happens on average substantially earlier in the fast races than in medium and slow pace races.

A notable aspect of this paper is the characterization of eventual medalist positioning at key tactical landmarks (e.g., the race kick; Figures 3A–C). Eventual gold and silver medalists were found to be positioned higher at the race kick in fast races than the bronze medalist (top 2 vs. top 4, respectively). In contrast, in slow races, unclear differences between eventual gold, silver and bronze medalists' position at the race kick, may reflect the fact that when the race is slow, everyone has increased probability of winning, as shown previously (Casado and Renfree, 2018). The present results build on the 2001–2012 analysis by Aragón et al. (2016) who found all race winners were in the top 4 when the finishing race kick was initiated, though these races were not categorized by pace category. Furthermore, Renfree et al. (2014) in a London 2012 Olympic case study, showed a correlation of ≥0.70 between a top 5 position with 300 m to go and finishing in a top 3 automatic qualifying position, representing a similar landmark to where the race kick occurred in Figure 2. Together these findings highlight the importance of enhancing in-race decision making (Hettinga et al., 2017) and preparation for multiple race scenarios (fast, medium, and slow) at championship events.

The present work offers several methodological advances in analysis of middle-distance tactical behavior. Understanding the nuance in tactical behavior between different race categories is limited within the scientific literature (Hanley et al., 2018), where race narratives often lie at the extremes (e.g., either one of “sit and kick,” or “gun to tape”; Mytton et al., 2015; Casado and Renfree, 2018). Our data shows there are important changes in tactical behavior (surge and position) as races become slower, creating deeper tactical insight and generating more avenues for further investigation. Additionally, through using expert observation in middle-distance running, we were able to provide greater resolution (within 10 m) on distances from the finish that surge and race kick events occurred, building on the one previous paper that has used resolution beyond 100 m sectors (Aragón et al., 2016). By adopting this novel observational method of analysis, we were able to illustrate corresponding positional changes of medal winning athletes at each surge and race kick event. This method allows deeper insight into within-race tactical behavior than that previously available and warrants further investigation across a wider cohort of event classifications and between genders.

Despite these advances to the literature, there are several methodological challenges that require consideration. For example, there is an assumption in our present methodology that all surges are the same (acceleration rate, repositioning of places due to pace gain etc.), which is a clear limitation. Further, our observational methods used only three expert judgements. We tempered any single sided bias by having multiple experts, and a second level verification, which whilst not objective, was performed by experienced practitioners (1st and third author). Human physical potential is sigmoidal (Tucker and Collins, 2012) and therefore, by nature of the study emphasis (European, Olympic and World Champions), there are a limited number of (a) races and (b) experts experienced in classifying tactical behavior of elite 1,500 m running. Creation of new methods (both manual observations and technology aided athlete tracking) to characterize such nuance would be beneficial in future studies, and would enable detailed tactical profiling of championship races and athletes.

## Practical Application

Within professional track and field racing, it can be a challenge for 1,500 m athletes to find quality races that are not set-up with a pace maker (e.g., Diamond league, European, and North American racing circuits) in order to prepare for major championship racing. This raises the question of how the tactical component in races with lots of surges and in-racing decision making i.e., slow or medium pace races (Figure 1) can be rehearsed ahead of championships. For example, perhaps tactical training session design or use of video analysis review to prepare athletes for a multitude of possible scenarios is an important consideration. Furthermore, developing athlete's affordance-based decision making to be resilient to uncontrollable opponent surges, could be a key competitive advantage. For example when the race starts out slow, athletes need to be prepared psychologically that there may be 1–2 surges every lap (Figure 1), and ultimately, the athlete needs to find confidence and flow to execute in a championship race. Top athletes must instinctively respond to, or dictate key tactical events. Nevertheless, current knowledge and rehearsal of tactical expectations in the practice arena, is perhaps an overlooked aspect of championship racing preparation.

## Conclusion

In agreement with previous tactical papers we highlight the clear role of the race kick in determining eventual championship race medalists, where, regardless of race category, the race kick occurred on the last lap. Importantly for the first time, three race categories across a continuum highlight substantial differences in surge events and tactical positioning before, and at initiation of the race kick. These new tactical insights have important implications for the tactical preparation of athletes and coaches for championship race execution.

## Data Availability Statement

The datasets for this manuscript are not publicly available because the data arose as a function of an employed position and therefore the rights to the data are owned by the employer at the time (High Performance Sport New Zealand) as Intellectual property.

## Author Contributions

GS, BD, and SR contributed to conception and design of the study, manuscript, figure and table revision, read and approved the submitted version. BD organized the database and produced first draft of the figures. GS and SR verified expert observation results. GS performed the statistical analysis and wrote the first draft of the manuscript.

### Conflict of Interest

The authors declare that the research was conducted in the absence of any commercial or financial relationships that could be construed as a potential conflict of interest.
